# Power Density Improvement of Piezoelectric Energy Harvesters via a Novel Hybridization Scheme with Electromagnetic Transduction

**DOI:** 10.3390/mi12070803

**Published:** 2021-07-07

**Authors:** Zhongjie Li, Chuanfu Xin, Yan Peng, Min Wang, Jun Luo, Shaorong Xie, Huayan Pu

**Affiliations:** 1 School of Mechatronic Engineering and Automation, Shanghai University, Shanghai 200444, China; lizhongjie@shu.edu.cn (Z.L.); xcfshu@shu.edu.cn (C.X.); luojun@shu.edu.cn (J.L.); srxie@shu.edu.cn (S.X.); phygood_2001@shu.edu.cn (H.P.); 2 Shanghai Institute of Intelligent Science and Technology, Tongji University, Shanghai 200092, China; 3 Engineering Research Center of Unmanned Intelligent Marine Equipment, Ministry of Education, 99 Shangda Rd., Shanghai 200444, China

**Keywords:** piezoelectric, electromagnetic, hybrid energy harvester, power density improvement

## Abstract

A novel hybridization scheme is proposed with electromagnetic transduction to improve the power density of piezoelectric energy harvester (PEH) in this paper. Based on the basic cantilever piezoelectric energy harvester (BC-PEH) composed of a mass block, a piezoelectric patch, and a cantilever beam, we replaced the mass block by a magnet array and added a coil array to form the hybrid energy harvester. To enhance the output power of the electromagnetic energy harvester (EMEH), we utilized an alternating magnet array. Then, to compare the power density of the hybrid harvester and BC-PEH, the experiments of output power were conducted. According to the experimental results, the power densities of the hybrid harvester and BC-PEH are, respectively, 3.53 mW/cm^3^ and 5.14 μW/cm^3^ under the conditions of 18.6 Hz and 0.3 g. Therefore, the power density of the hybrid harvester is 686 times as high as that of the BC-PEH, which verified the power density improvement of PEH via a hybridization scheme with EMEH. Additionally, the hybrid harvester exhibits better performance for charging capacitors, such as charging a 2.2 mF capacitor to 8 V within 17 s. It is of great significance to further develop self-powered devices.

## 1. Introduction

The extensive use of smart sensor devices (such as marine environment monitoring wireless sensors, wildfire detectors, etc.), which are usually powered by batteries, leads to a large amount of power consumption. However, due to the limited lifespan of batteries, it severely restricts the continuous operation of smart sensor devices and replacing batteries regularly can cause high costs. To solve these problems, developing self-powered devices by using some approaches to harvest ambient energy, for instance, solar energy [1,2,3], wind [4,5,6], tidal energy [7], thermal energy [8,9], mechanical vibration [10,11], and pyroelectric energy [12,13], is a feasible program. The commonly used energy harvesting mechanism is piezoelectric [14,15,16,17].

The piezoelectric energy conversion mechanism has been used to extract kinetic energy because of the high power density, simple operation mechanism, and design flexibility [18]. The basic configuration of PEH is composed of a mass block, a piezoelectric patch, and a cantilever beam. Based on the basic configuration, many novel designs were proposed. Zhang et al. [19] designed a multi-impact harvester with superior performance to extract energy under the low-frequency vibration. Iman et al. [20] collected energy from human motion by using a harvester and powered electronic devices. Based on impact vibration, a low-frequency PEH, assembled with two rigid generating beams and a compliant driving beam, was presented by Gu, which can achieve an average power of 1.53 mW under the conditions of 20.1 Hz and 0.4 g [21]. Besides, some original designs of PEHs were proposed, including a cantilever PEH with a revolute joint [22], a press-button type PEH [23], a PEH with a sandwich structure [24], a T-shaped PEH with internal resonance [25], a truss-based compressive-mode PEH [26], a PEH based on the piezoelectric stack [27,28], and a U-shaped bi-directional PEH [29].

Recently, piezoelectric-electromagnetic hybrid harvesters have attracted gravitational attention [30]. Iqbal et al. [31] presented a hybrid harvester, which contains the PEH and EMEH, to collect low-frequency vibration energy from walking motion. The harvester can generate the power of 51 μW and 36 μW from EMEH and PEH, respectively. Iqbal et al. [32] designed a multimodal hybrid bridge harvester. The power of 2214.32 μW and 155.7 μW were, respectively, generated from its electromagnetic and piezoelectric portions. Edwards et al. [33] introduced a novel low-frequency vibration energy harvester and conducted a series of simulations and experiments. Based on the experimental results, the harvester can, respectively, achieve the average power of 46.2 μW and 3.6 μW from electromagnetic and piezoelectric transducers at 5 Hz. Pyo et al. [34] investigated a hybrid harvester by using frequency up-conversion, which can extract energy from an extremely low-frequency mechanical motion and generate peak power of 6.03 mW and 1.35 mW from electromagnetic and piezoelectric portions, respectively.

In the above work, researchers mostly use a magnet or a simple combination of several magnets to form the hybrid energy harvester, which can result in small output power of EMEHs. Additionally, Li et al. [35] have proven that an alternating magnet array, which can cause abrupt magnetic flux density changes, can improve the output power of EMEHs. Therefore, we proposed a novel hybrid scheme with an alternating magnet array to improve the power density of PEHs.

In this paper, we presented a hybrid harvester based on the BC-PEH, which displayed excellent performance under the weak excitation. The key contributions include: first, designing a novel hybrid scheme to improve the power density of BC-PEH; second, implementing a simulation to testify the abrupt magnetic flux density (MFD) changes caused by an alternating magnet array; third, conducting experiments of open-circuit voltage, frequency sweep, output power, and charging capacitors to compare the output performance of the hybrid harvester and the BC-PEH; and finally, further demonstrating the high power density of the hybrid harvester by charging millifarad level capacitors.

## 2. Configuration and Simulation

Based on the BC-PEH composed of a mass block, a piezoelectric patch, and a cantilever beam, we replaced the mass block by a magnet array and added a coil array to form the hybrid energy harvester (PEH source and EMEH source). Due to the magnetic damping, the deformation of the piezoelectric material becomes small, so the output power of the PEH decreases. However, the EMEH generates power in the reciprocating motion of the magnet array. Therefore, to compare the total output power of the hybrid harvester and the BC-PEH, we made a prototype and conducted experiments of open-circuit voltage, frequency sweep, output power, and charging capacitors.

**Figure 1:**
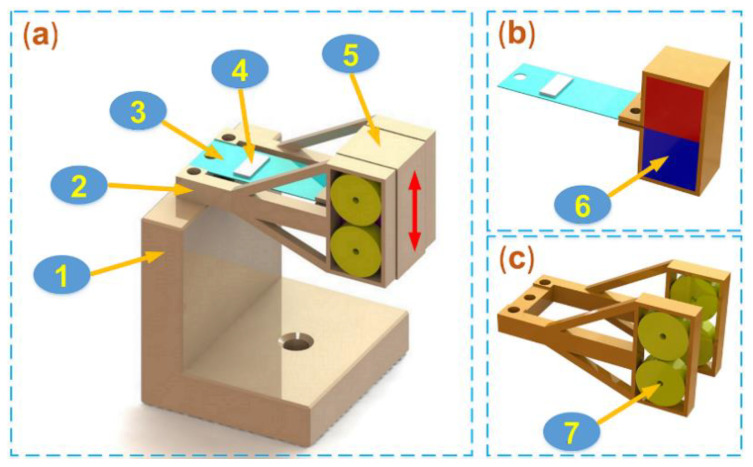
The configuration of the hybrid harvester. The components are, respectively: 1-base; 2-coil bracket; 3-cantilever beam; 4-piezoelectric patch; 5-magnet frame; 6-magnet; and 7-coil. (**a**) The configuration. (**b**) The magnet array. (**c**) The coil array.

The configuration of the hybrid harvester was illustrated in Figure 1a. It is composed of a base, a cantilever beam, a coil bracket, and a magnet frame. In Figure 1a, one end of the cantilever beam and the coil bracket are fixed to the base, and the magnet frame is connected with the other end of the cantilever beam. As shown in Figure 1b, the magnet array contains two magnets, which are installed in the magnet frame, and a proof mass is constituted of the magnet array and magnet frame. As shown in Figure 1c, the coil array placed on either side of the magnet array, including four coils, is mounted in the coil bracket. An electromagnetic energy conversion mechanism is constructed by the coil array and proof mass. Additionally, a piezoelectric patch (as shown in Figure 1a) acts as a piezoelectric energy conversion mechanism with the cantilever beam and proof mass.

**Figure 2:**
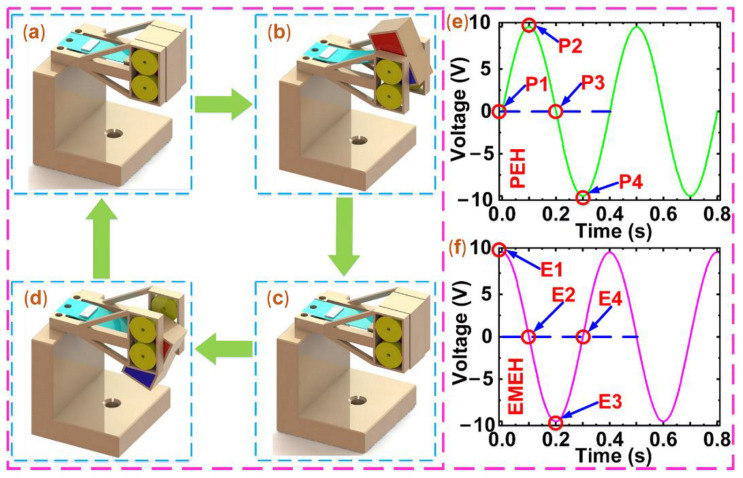
The positions of the cantilever beam in the reciprocating motion of the proof mass. (**a**) The initial position. (**b**) The position of maximum upward displacement. (**c**) The initial position. (**d**) The position of maximum downward displacement. (**e**) The simulated voltage of the PEH. (**f**) The simulated voltage of the EMEH.

When the hybrid harvester is applied excitations, the proof mass is forced to reciprocate in the indicated direction in Figure 1a, which causes the cantilever beam to bend. Then, the bending induces the piezoelectric patch to deform and leads to strain generation inside the material. Therefore, it yields the voltage according to the direct piezoelectric effect. At the same time, relative movement occurs between the proof mass and the coil array. Therefore, the coils generate an induced electromotive force ($E_{mf}$), which can be gauged by using Equation (1).

(1) $$E_{mf}=-N\frac{d(BS)}{d(t)}$$

where, N, B, S, and t are the turns of the coil array, the MFD, the area of the coil, and the time, respectively. In the reciprocating motion of the proof mass, the cantilever beam firstly goes from the initial position to the point of maximum upward displacement, then starts moving down, then reaches the position of maximum downward displacement, and then returns to the initial position. As shown in Figure 2a,c, the cantilever beam is at the initial position and does not deform, so the output voltage of the PEH is zero, which is found in Figure 2e (P1 and P3). However, the proof mass gets the largest movement speed at this moment, so the maximum instantaneous voltage is obtained from the EMEH according to Equation (1), as shown in Figure 2f (E1 and E3). Figure 2b,d show the cantilever beam is at the positions of maximum upward displacement and maximum downward displacement. At this time, the piezoelectric patch achieves the maximum deformation, so the PEH yields the maximum output voltage, as shown in Figure 2e (P2 and P4). Besides, the movement speed of the proof mass is zero, so the EMEH does not generate the output voltage, as shown in Figure 2f (E2 and E4).

**Figure 3:**
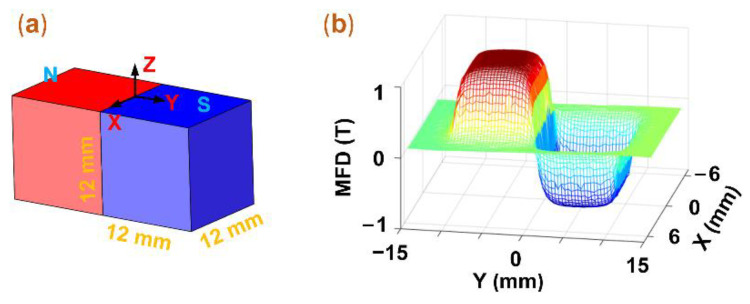
The simulation of the MFD. (**a**) the simulation model; (**b**) the MFD distribution in the Z direction of the model.

According to Equation (1), we can enhance the output voltages of EMEHs by increasing the change rate of the MFD. Moreover, the alternating magnet array can cause abrupt magnetic flux density changes. Therefore, we utilize the way of alternating arrangement of magnetic poles in the scheme. According to the model shown in Figure 3a, the simulation was conducted to demonstrate this phenomenon mentioned above by using COMSOL Multiphysics 5.4 (Sweden). First, based on Figure 3a, we constructed two cube magnets with a length of 12 mm in COMSOL. The magnetic flux (1 T) directions of the two magnets are opposite along the Z-axis of the coordinate system. Then, the MFD distribution was depicted in the Z direction of the model. Figure 3b displays a step change in MFD between two continuous magnets. Namely, the MFD immediately decreases from the peak value to the minimal value, which verifies the phenomenon above. Moreover, the maximum value of the MFD is on the polar surface of magnets, so the distance between coils and magnets should be as small as possible.

## 3. Experiment and Discussion

### 3.1. Prototype Fabrication and Experiment Setup

**Figure 4:**
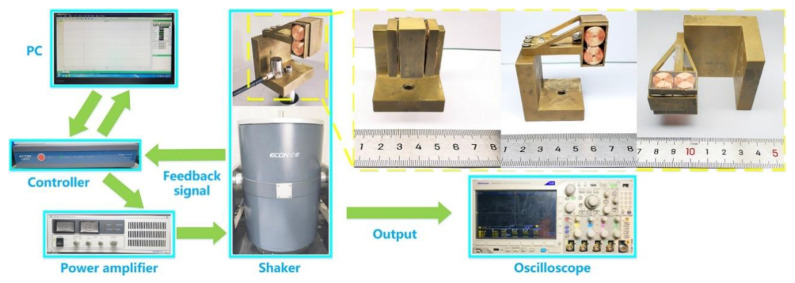
The prototype and experiment conditions.

According to the configuration in Figure 1a, a prototype was fabricated and the experiments of open-circuit voltage, frequency sweep, output power, and charging capacitors were conducted to examine the output performance of the prototype and compare the outputs with/without the hybridization scheme. The prototype and experiment conditions are shown in Figure 4. The coil bracket and one end of the cantilever beam were fixed to the base with screws, and the magnet frame was joined to the other end of the cantilever beam with screws and nuts. The base, cantilever beam, coil bracket, and magnet frame of the hybrid energy harvester were all made of copper. Furthermore, the distance between the coil and the magnet was set to 0.5 mm.

The types of the shaker, accelerometer, controller, power amplifier, and oscilloscope, which were used in the experiment, are Econ E-JZK-50, Econ EA-YD-181, Econ VT-9002, Econ E5874A (ECON, Kunshan, China) and Tektronix mdo3024 (Tektronix, OK, USA), respectively. The prototype was fixed on the shaker with a screw, and the accelerometer was fixed on the base by using tape. Then, the relevant parameters were set on the PC, and the experiments of open-circuit voltage, frequency sweep, output power, and charging capacitors were conducted. The control signal was generated by the controller after receiving the instructions from the PC. When the power amplifier got the control signal from the controller, the shaker was driven. Then, the feedback signal was transmitted to the controller by the accelerometer, so the hybrid energy harvester works under a constant acceleration. The output voltages of the hybrid energy harvester were all measured by a digital oscilloscope. The detailed material properties and geometric parameters of the prototype are shown in Table 1.

### 3.2. Experiments of Open-Circuit Voltage

**Figure 5:**
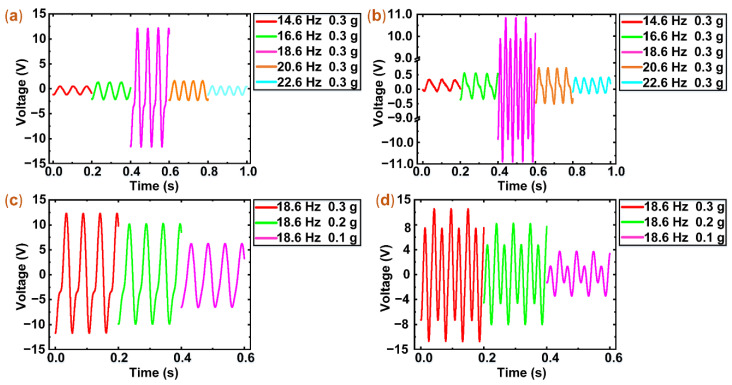
The output voltages of the PEH and EMEH under different frequencies and acceleration. (**a**,**b**) The output voltages of the PEH and EMEH under different frequencies (14.6 Hz, 16.6 Hz, 18.6 Hz, 20.6 Hz, and 22.6 Hz) and constant acceleration (0.3 g). (**c**,**d**) The output voltages of the PEH and EMEH under constant frequency (18.6 Hz) and different acceleration (0.1 g, 0.2 g, and 0.3 g).

The open-circuit voltages of the hybrid energy harvester (PEH source and EMEH source) were measured under the harmonic excitation of different frequencies (14.6 Hz, 16.6 Hz, 18.6 Hz, 20.6 Hz, and 22.6 Hz) and constant acceleration (0.3 g). The open-circuit voltages of the PEH and EMEH are shown in Figure 5a,b.

In Figure 5a, a peak-to-peak open-circuit voltage of the PEH is 2.0 V under the excitation frequency of 14.6 Hz. The voltage increases with the rise of excitation frequency and reaches a maximum of 25.3 V at 18.6 Hz. Then, the voltage decreases with the increase of excitation frequency and the voltage is 1.9 V at 22.6 Hz. As shown in Figure 5b, the voltage change of the EMEH is similar to that of the PEH. The voltage is 0.58 V at 14.6 Hz. As the excitation frequency increases to 18.6 Hz, the voltage reaches a peak of 21.9 V. Then, when the excitation frequency is 22.6 Hz, the voltage decreases to 0.74 V.

The voltage changes of the PEH and EMEH are the same behavior pattern. From 14.6 Hz to 18.6 Hz, the voltage continually increases. The voltage changes of the frequencies from 14.6 Hz to 16.6 Hz are less obvious than that of the frequencies from 16.6 Hz to 18.6 Hz. From 18.6 Hz to 22.6 Hz, the voltages unremittingly decrease. The voltage changes of the frequencies from 18.6 Hz to 20.6 Hz are more observable than that of the frequencies from 20.6 Hz to 22.6 Hz. When the excitation frequency increases to near the resonant frequency, the maximum deformation of the piezoelectric patch and the peak rate of magnetic flux density change is achieved. Therefore, the voltages of the PEH and EMEH display an obvious change.

To further study open-circuit voltages of the PEH and EMEH, we measured the voltages of the PEH and EMEH under constant frequency (18.6 Hz) and different excitation acceleration (0.1 g, 0.2 g, 0.3 g). In Figure 5c, the voltage of the PEH is 13.01 V at 0.1 g. As the excitation acceleration rises, the voltage increases. When the excitation acceleration is 0.2 g and 0.3 g, the voltages are 20.65 V and 25.73 V, respectively. As shown in Figure 5d, the voltage trend of the EMEH and the PEH is similar. When the excitation acceleration is 0.1 g, 0.2 g, and 0.3 g, the voltages are 7.34 V, 16.47 V, and 21.97 V, respectively.

In Figure 5, the open-circuit voltage waveform of the PEH and EMEH is not a standard harmonic. They, respectively, show resembling sawtooth waves and alternating sine waves of different amplitude in the resonant region. At resonance, the deformation of the cantilever beam is large and non-linear, which results in the strain of the piezoelectric patch and the displacement change of the magnet array being non-linear. Therefore, the voltage waveform of the PEH and EMEH displays a nonstandard sine waveform.

### 3.3. Experiments of Frequency Sweep

**Figure 6:**
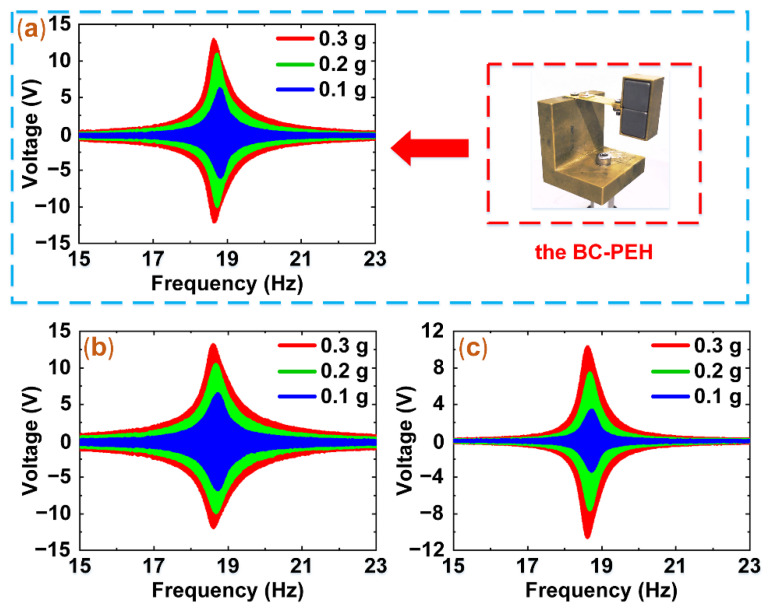
The output voltages of the BC-PEH and the hybrid harvester under different acceleration (0.1 g, 0.2 g, and 0.3 g). (**a**) The output voltages of the BC-PEH. (**b**) The output voltages of the PEH. (**c**) The output voltages of the EMEH.

Afterward, to compare the output voltages of the BC-PEH and the hybrid harvester, the frequency-sweep experiments were conducted. In these experiments, the sweep rate and frequency domain are 0.1 Hz/s and (15 Hz, 23 Hz), respectively. Based on the different acceleration (0.1 g, 0.2 g, and 0.3 g), the open-circuit voltages of the BC-PEH and hybrid harvester (PEH source and EMEH source) were measured. The experimental results are shown in Figure 6.

Figure 6a–c display the resonant frequency (18.6 Hz) of the prototype. The voltages of the BC-PEH and hybrid harvester vary as the excitation acceleration increases and reach a maximum at 18.6 Hz. The voltages of the PEH are 25.7 V, 20.6 V, and 13.1 V under the resonant frequency and acceleration of 0.3 g, 0.2 g, and 0.1 g, respectively. The behavior patterns of the voltage changes of the BC-PEH and EMEH are similar to that of the PEH. The voltages of the BC-PEH are 25.2 V, 21.1 V, and 12.3 V under the acceleration of 0.3 g, 0.2 g, and 0.1 g, respectively. Similarly, the voltage changes of 21.9 V, 16.5 V, and 7.4 V for EMEH are obtained under the acceleration of 0.3 g, 0.2 g, and 0.1 g, respectively.

In the sweep experiments, the current value of the EMEH is approximately 0, so the damping of the BC-PEH and hybrid harvester mainly comes from the mechanical damping. Therefore, as shown in Figure 6a,b, the voltage values of the BC-PEH and PEH are nearly the same.

### 3.4. Experiments of Output Power

**Figure 7:**
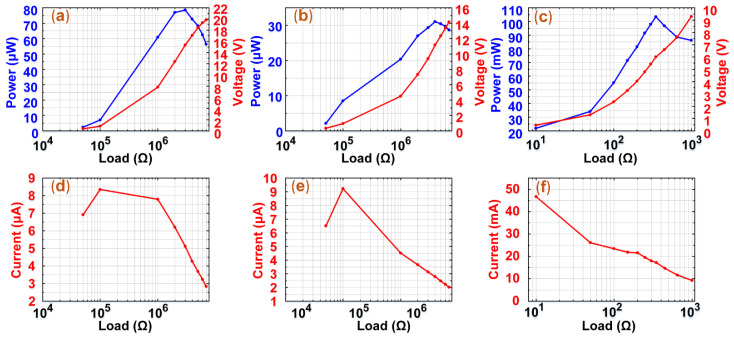
The output power, voltages, and currents of the BC-PEH and hybrid harvester. (**a**–**c**) The peak-to-peak output power and voltages of the BC-PEH, PEH, and EMEH. (**d**–**f**) The peak-to-peak output currents of the BC-PEH, PEH, and EMEH.

To compare the output performance of the BC-PEH and hybrid harvester, the output power experiments were carried out under the conditions of 0.3 g and 18.6 Hz. In these experiments, we recorded the power, voltages, and currents of the BC-PEH and hybrid harvester (PEH source and EMEH source). The results are shown in Figure 7.

Figure 7b,e show the changes in the power, voltages, and currents of the PEH under different external electric resistances. The power of the PEH gradually increases and reaches a maximum of 31.08 μW and then decreases. The voltages of the PEH continue to enlarge until it approaches the open-circuit voltage as the external electric resistances increase. In addition, the currents of the PEH slightly rise and then continuously decline. Figure 7a,c,d,f show the changes of the power, voltages, and currents of the BC-PEH and EMEH, which are similar to that of the PEH. The maximum power of the BC-PEH and EMEH is 78.5 μW and 103.5 mW, respectively. Compared to the power density of 5.14 μW/cm^3^ of the BC-PEH, the power density of 3.53 mW/cm^3^ of the hybrid harvester is 686 times as high as the BC-PEH. Besides, it should be mentioned that the peak power of the BC-PEH is higher than the PEH, which is attributed to the electromagnetic damping.

To further explore the ability of the hybrid harvester as a power source to supply power to an external load, the average power ($P_{avg}$) of the hybrid harvester was calculated. The voltage waveform of the BC-PEH and hybrid harvester is a nonstandard sine waveform, so we use Equation (2) to obtain the average power of the BC-PEH and hybrid harvester. The average power of the hybrid harvester is approximately 7.98 mW and the BC-PEH is 4.38 μW. Therefore, the average power of the hybrid harvester is 1821 times as high as the BC-PEH.

(2) $$P_{avg}=\int_{0}^{t}U^{2}(t)/R_{m}dt/T$$

where U(t), $R_{m}$, $P_{avg}$, and T are the instantaneous voltage, matching impedance, average power, and full-time of an excitation, respectively.

In Table 2, we displayed a performance comparison for the proposed prototype and other hybrid harvesters with different configurations.

The power density of the prototype can reach 3.53 mW/cm^3^ under a weak acceleration (0.3 g) in this paper, which is 14 times, 7 times, and 4 times as high as Refs [34,38,42]. Therefore, the output performance of the prototype in this paper is superior. 

### 3.5. Experiments of Charging Capacitors

**Figure 8:**
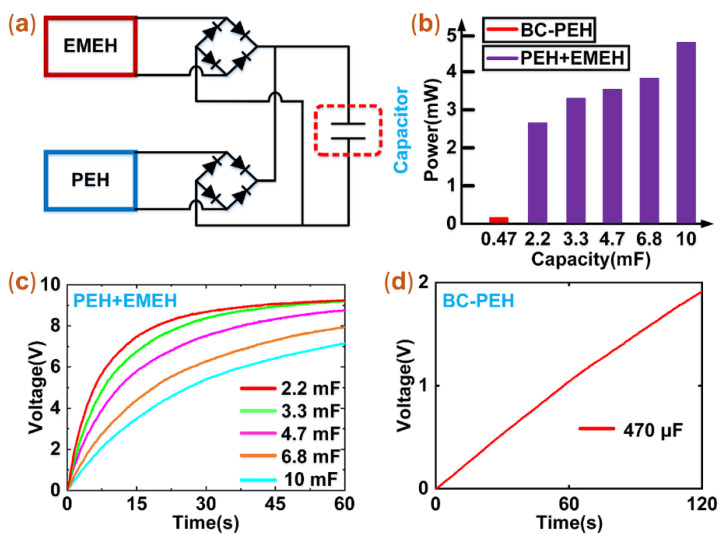
The experimental circuit and results of charging capacitors. (**a**) The circuit of charging capacitors. (**b**) The histogram of the average charging power. (**c**) The results of charging capacitors by using the hybrid harvester. (**d**) The result of charging capacitor by using the BC-PEH.

To further compare the output power of the BC-PEH and hybrid harvester, we conducted charging experiments. We firstly designed an experimental circuit, which contains two harvesters and rectifiers, as shown in Figure 8a. Since the open-circuit voltage magnitudes of the PEH and EMEH are at the same level, we chose to connect the PEH and EMEH in parallel. Two rectifiers were, respectively, connected to the ends of the PEH and EMEH and converted the negative current signals of the PEH and EMEH into positive ones.

In the charging experiments, we selected six capacitors: 470 μF, 2.2 mF, 3.3 mF, 4.7 mF, 6.8 mF, and 10 mF, and set the experimental conditions as follows: a constant acceleration of 0.3 g, a constant frequency of 18.6 Hz. We used the BC-PEH and the hybrid harvester to conduct comparative charging experiments. Before experiments, we fully discharged the capacitors. For the capacitor of 470 μF, it was only charged to 1.95 V within 120 s by using the BC-PEH, as shown in Figure 8d. When we used the hybrid harvester to charge the capacitors of 2.2 mF, 3.3 mF, 4.7 mF, 6.8 mF, and 10 mF, they were, respectively, charged to 8 V, 7.2 V, 6.3 V, 4.9 V, and 4.1 V within 17 s, as shown in Figure 8c. Compared to the BC-PEH, the hybrid harvester can charge a larger capacitor to a higher voltage in a shorter period. The hybrid harvester exhibits excellent charge performance.

It should be mentioned that the average power ($P_{a}$) for charging capacitors can be calculated by Equation (3):

(3) $$P_{a}=C_{p}(U_{2}{}^{2}-U_{1}{}^{2})/2\Delta t$$

where $P_{a}$, $C_{P}$, $U_{1}$, $U_{2}$ and Δt represent the average charging power, capacity, initial voltage, final voltage, and charging time, respectively. To compare the power of the BC-PEH and the hybrid harvester, we made a histogram of the power, as shown in Figure 8b. When the capacitor of 10 mF was charged, the power of the hybrid harvester reached 4.94 mW. Compared to the power of 0.0147 mW for charging the capacitor of 470 μF by using the BC-PEH, the average charging power of the hybrid harvester is 336 times higher than that of the BC-PEH. In addition, to store more energy at the same time, it is necessary to choose a capacitor with a large capacity.

## 4. Conclusions

This paper proposed a novel hybridization scheme with electromagnetic transduction to enhance the power density of PEHs. The hybrid energy harvester was designed based on the BC-PEH. To compare the power density of the BC-PEH and the hybrid energy harvester, we built a prototype and conducted experiments of open-circuit voltage, frequency sweep, output power, and charging capacitors. According to the experimental results, the key conclusions of this paper are as follows:

1. The EMEH can yield a high voltage of 21.9 V under a weak acceleration of 0.3 g by using an alternating magnet array, which can result in abrupt magnetic flux density changes.
2. Comparing the peak power of the BC-PEH and hybrid harvester, the output power (103.53 mW) of the hybrid harvester is 1318 times as high as the output power (78.5 μW) of the BC-PEH.
3. Comparing the power densities and average power of the BC-PEH and hybrid harvester, the power density and average power of the hybrid harvester are, respectively, 686 times and 1821 times higher than that of the BC-PEH.
4. The hybrid harvester also displays excellent charging performance because of the high output power. According to the experimental results, the average charging power of the hybrid harvester is 336 times higher than that of the BC-PEH.

The hybrid energy harvester shows a better energy capture performance, which verifies that the power density improvement of PEHs can use a hybridization scheme with electromagnetic transduction and also displays its great potential to successfully power low-power electronic components.

## Figures and Tables

**Table 1:** Material properties and the geometric parameters of the prototype.

Description		Value
Prototype	Dimensions (mm^3^)	49 × 23 × 26
Cantilever beam	Dimensions (mm^3^)	35 × 12 × 0.5
Magnet array	Number	1
	Number of magnets	2
Magnet	Dimensions (mm^3^)	12 × 12 × 12
	Magnet grade	N52
	Material	NdFeB
Coil array	Number	2
	Number of coils	4
Coil	Outside dimension (mm^3^)	12 × 4.7
	Inside dimension (mm^3^)	1.8 × 4.7
	Number of turns	1860
	Resistance (Ω)	85.3
	Wire diameter (mm)	0.1
Piezoelectric patch	Piezoelectric material	PZT-5H
	Dimensions (mm^3^)	7.5 × 3.5 × 0.5

**Table 2:** The performance comparison of hybrid harvesters.

Ref.	Dimensions (cm^3^)	Frequency (Hz)	Excitation (Speed or Acceleration)	Power (mW)	Power Density (mW/cm^3^)
[36]	105 × 30 × 20	17	0.4 g	15.82	0.251
[37]	3.85 × 3.4 × 3.7	/	4–6 km/h	0.55	1.14 × 10^−3^
[38]	46.8	51	0.5 g	1.67	0.036
[39]	1.84	2	/	0.0298	0.01619
[40]	5	23.3	0.4 g	2.26	0.452
[41]	10 × 4 × 0.1	33.5	0.3 g	3.32	0.83
[42]	4 × 1.5 × 4	113.5	0.6 g	3.54	0.1475
[43]	19.2	5.2	2 g	1.2288	0.064
[44]	4 × 4 × 1	/	1 m/s	14.0135	0.876
[45]	6 × 0.7 × 2	16.8	0.5 g	3.12	0.371
This paper	4.9 × 2.3 × 2.6	18.6	0.3 g	103.51	3.53

## Data Availability

The data supporting the findings of this paper is available from the corresponding authors on request.
